# Sequencing by avidity enables high accuracy with low reagent consumption

**DOI:** 10.1038/s41587-023-01750-7

**Published:** 2023-05-25

**Authors:** Sinan Arslan, Francisco J. Garcia, Minghao Guo, Matthew W. Kellinger, Semyon Kruglyak, Jake A. LeVieux, Adeline H. Mah, Haosen Wang, Junhua Zhao, Chunhong Zhou, Andrew Altomare, John Bailey, Matthew B. Byrne, Chiting Chang, Steve X. Chen, Byungrae Cho, Claudia N. Dennler, Vivian T. Dien, Derek Fuller, Ryan Kelley, Omid Khandan, Michael G. Klein, Michael Kim, Bryan R. Lajoie, Bill Lin, Yu Liu, Tyler Lopez, Peter T. Mains, Andrew D. Price, Samantha R. Robertson, Hermes Taylor-Weiner, Ramreddy Tippana, Austin B. Tomaney, Su Zhang, Minna Abtahi, Mark R. Ambroso, Rosita Bajari, Ava M. Bellizzi, Chris B. Benitez, Daniel R. Berard, Lorenzo Berti, Kelly N. Blease, Angela P. Blum, Andrew M. Boddicker, Leo Bondar, Chris Brown, Chris A. Bui, Juan Calleja-Aguirre, Kevin Cappa, Joshua Chan, Victor W. Chang, Katherine Charov, Xiyi Chen, Rodger M. Constandse, Weston Damron, Mariam Dawood, Nicole DeBuono, John D. Dimalanta, Laure Edoli, Keerthana Elango, Nikka Faustino, Chao Feng, Matthew Ferrari, Keith Frankie, Adam Fries, Anne Galloway, Vlad Gavrila, Gregory J. Gemmen, James Ghadiali, Arash Ghorbani, Logan A. Goddard, Adriana Roginski Guetter, Garren L. Hendricks, Jendrik Hentschel, Daniel J. Honigfort, Yun-Ting Hsieh, Yu-Hsien Hwang Fu, Scott K. Im, Chaoyi Jin, Shradha Kabu, Daniel E. Kincade, Shawn Levy, Yu Li, Vincent K. Liang, William H. Light, Jonathan B. Lipsher, Tsung-li Liu, Grace Long, Rui Ma, John M. Mailloux, Kyle A. Mandla, Anyssa R. Martinez, Max Mass, Daniel T. McKean, Michael Meron, Edmund A. Miller, Celyne S. Moh, Rachel K. Moore, Juan Moreno, Jordan M. Neysmith, Cassandra S. Niman, Jesus M. Nunez, Micah T. Ojeda, Sara Espinosa Ortiz, Jenna Owens, Geoffrey Piland, Daniel J. Proctor, Josua B. Purba, Michael Ray, Daisong Rong, Virginia M. Saade, Sanchari Saha, Gustav Santo Tomas, Nicholas Scheidler, Luqmanal H. Sirajudeen, Samantha Snow, Gudrun Stengel, Ryan Stinson, Michael J. Stone, Keoni J. Sundseth, Eileen Thai, Connor J. Thompson, Marco Tjioe, Christy L. Trejo, Greg Trieger, Diane Ni Truong, Ben Tse, Benjamin Voiles, Henry Vuong, Jennifer C. Wong, Chiung-Ting Wu, Hua Yu, Yingxian Yu, Ming Yu, Xi Zhang, Da Zhao, Genhua Zheng, Molly He, Michael Previte

**Affiliations:** Element Biosciences, San Diego, CA USA

**Keywords:** Next-generation sequencing, Transcriptomics

## Abstract

We present avidity sequencing, a sequencing chemistry that separately optimizes the processes of stepping along a DNA template and that of identifying each nucleotide within the template. Nucleotide identification uses multivalent nucleotide ligands on dye-labeled cores to form polymerase–polymer–nucleotide complexes bound to clonal copies of DNA targets. These polymer–nucleotide substrates, termed avidites, decrease the required concentration of reporting nucleotides from micromolar to nanomolar and yield negligible dissociation rates. Avidity sequencing achieves high accuracy, with 96.2% and 85.4% of base calls having an average of one error per 1,000 and 10,000 base pairs, respectively. We show that the average error rate of avidity sequencing remained stable following a long homopolymer.

## Abstract (web summary)

A sequencing chemistry that separates nucleotide identification from nucleotide incorporation achieves high accuracy.

## Main

Avidity sequencing chemistry enables a diversity of applications that include single-cell RNA sequencing (RNA-seq) and whole-human-genome sequencing. For the human sample HG002, avidity sequencing reached a single-nucleotide polymorphism (SNP) F1 score of 0.9958 and small-indel F1 score of 0.9954.

Over the past 15 years, highly parallel sequencing methods have enabled a broad set of applications^1–8^. Multiple technologies have been introduced during this time, each having various strengths and limitations^9^. The technologies vary by accuracy, read length, run time and cost. The most widely used method uses highly parallel and accurate short-read sequencing, described in ref. ^10^ and termed sequencing by synthesis (SBS).

The SBS methodology sequences DNA by controlled (that is, one at a time) incorporation of modified nucleotides^11^. The modifications consist of a 3′ blocking group and a dye label^12,13^. The blocking group ensures that only a single nucleotide is incorporated, and the dye label enables identification of each nucleotide following an imaging step. The blocking group and label are subsequently removed, completing the sequencing cycle. The cycle is repeated with the incorporation of the next blocked and labeled nucleotide. Incorporation of the modified nucleotide meets two objectives: to advance the polymerase along the DNA template and to differentially label the incorporated nucleotide for base identification. Although combination of the two processes is efficient, it prevents independent optimization of the processes. High-yielding and rapid incorporation requires micromolar concentrations of nucleotides to drive the polymerizing reaction^14–18^. The alternative, of allowing longer incorporation times, results in longer cycle times that have an additive effect over 300 cycles of stepwise sequencing.

We present a different sequencing chemistry, termed avidity sequencing, that separates and independently optimizes the controlled incorporation and nucleotide identification steps to achieve increased base-calling accuracy relative to SBS while reducing the concentration of key reagents to nanomolar scale. To advance this approach, we first had to overcome the technical challenge of signal persistence. For example, a potential strategy for separation of the steps described above could be to first incorporate a 3′ blocked but unlabeled nucleotide and then to bind a complementary labeled nucleotide to the subsequent base in the template for base identification. This approach is problematic because the dissociation rate for single nucleotides from a polymerase–template complex is large, and the polymerase–nucleotide complex does not remain stable throughout imaging unless prohibitively high concentrations of nucleotides are present in the bulk solution. To overcome this challenge, we used avidity.

**Fig. 1:**
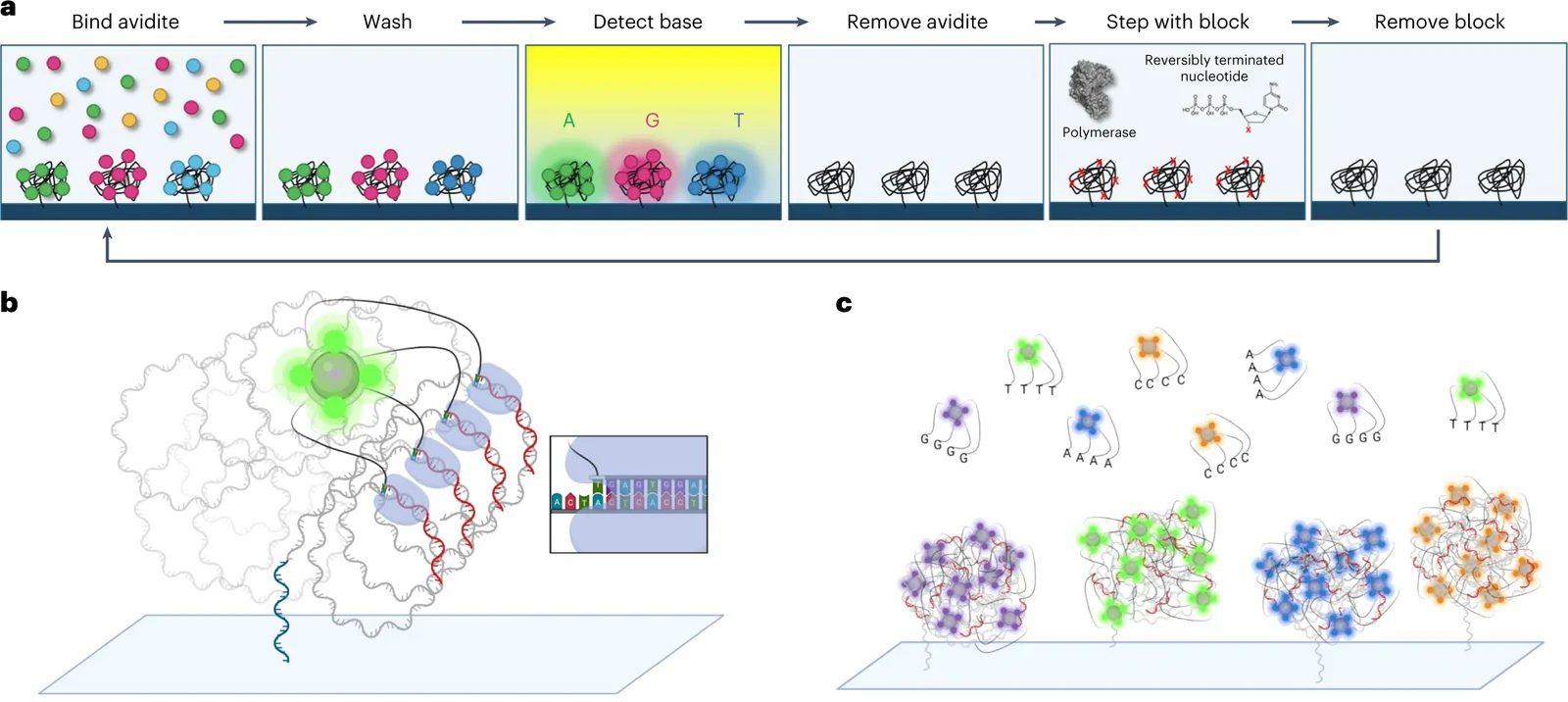
Avidity sequencing workflow and scheme. **a**, Sequencing by avidity. A reagent containing multivalent avidite substrates and an engineered polymerase are combined with DNA polonies inside a flowcell. The engineered polymerase binds to the free 3′ ends of the primer-template of a polony and selects the correct cognate avidite via base-pairing discrimination. The multivalent avidite interacts with multiple polymerases on one polony to create avidity binding that reduces the effective *K*_d_ of the avidite substrates 100-fold compared with a monovalent dye-labeled nucleotide, allowing productive binding of nanomolar concentrations. Multiple polymerase-mediated binding events per avidite ensure a long signal persistence time. Imaging of fluorescent, bound avidites enables base classification. Following detection, avidites are removed from the polonies. Extension by one base using an engineered polymerase incorporates an unlabeled, blocked nucleotide. A terminal 3′ hydroxyl is regenerated on the DNA strand, allowing repetition of the cycle. **b**, Rendering of a single avidite bound to a DNA polony via polymerase-mediated selection. The initial surface primer used for library hybridization and extension during polony formation is shown in blue. Sequencing primers (red) are shown annealed to the single-strand DNA polony (gray). Each arm of the avidite (black) connects the avidite core containing multiple fluorophores (green) to a nucleotide substrate. The polymerase bound to the sequencing primer selects the correct nucleotide to base pair with the templating base (inset). The result is multiple base-mediated anchor points noncovalently attaching the avidite to the DNA polony. **c**, Rendering of multiple DNA polonies with template-specific avidites bound during the binding step of the cycle (polymerase not shown for simplicity). Many avidites bind to each DNA polony generating a fluorescent signal during detection. Multiple long, flexible polymer linkers connect the core to the nucleotide substrates.

Avidity refers to the accumulated strength of multiple affinities of individual noncovalent binding interactions, which can be achieved when multivalent ligands tethered in close proximity simultaneously bind to their targets^19^. Coincident binding increases ligand affinity and residence time^20^. As an example of the potential impact of avidity on both affinity and decreased dissociation rate, Zhang et al.^21^ demonstrated that, by changing a monomeric to a pentameric nanobody, it is possible to decrease dissociation rates by three to four orders of magnitude. Our approach was to use avidity for nucleotide detection within the sequencing chemistry (Fig. 1). We demonstrate here that avidity sequencing achieves accuracy, surpassing an average of one error per 10,000 base pairs (bp) (*Q*40), and enables a diversity of applications that include single-cell RNA-seq and whole-human-genome sequencing. We also demonstrate an improved ability of this chemistry to sequence through homopolymer sequences.

## Results

**Extended Data Fig. 1:**
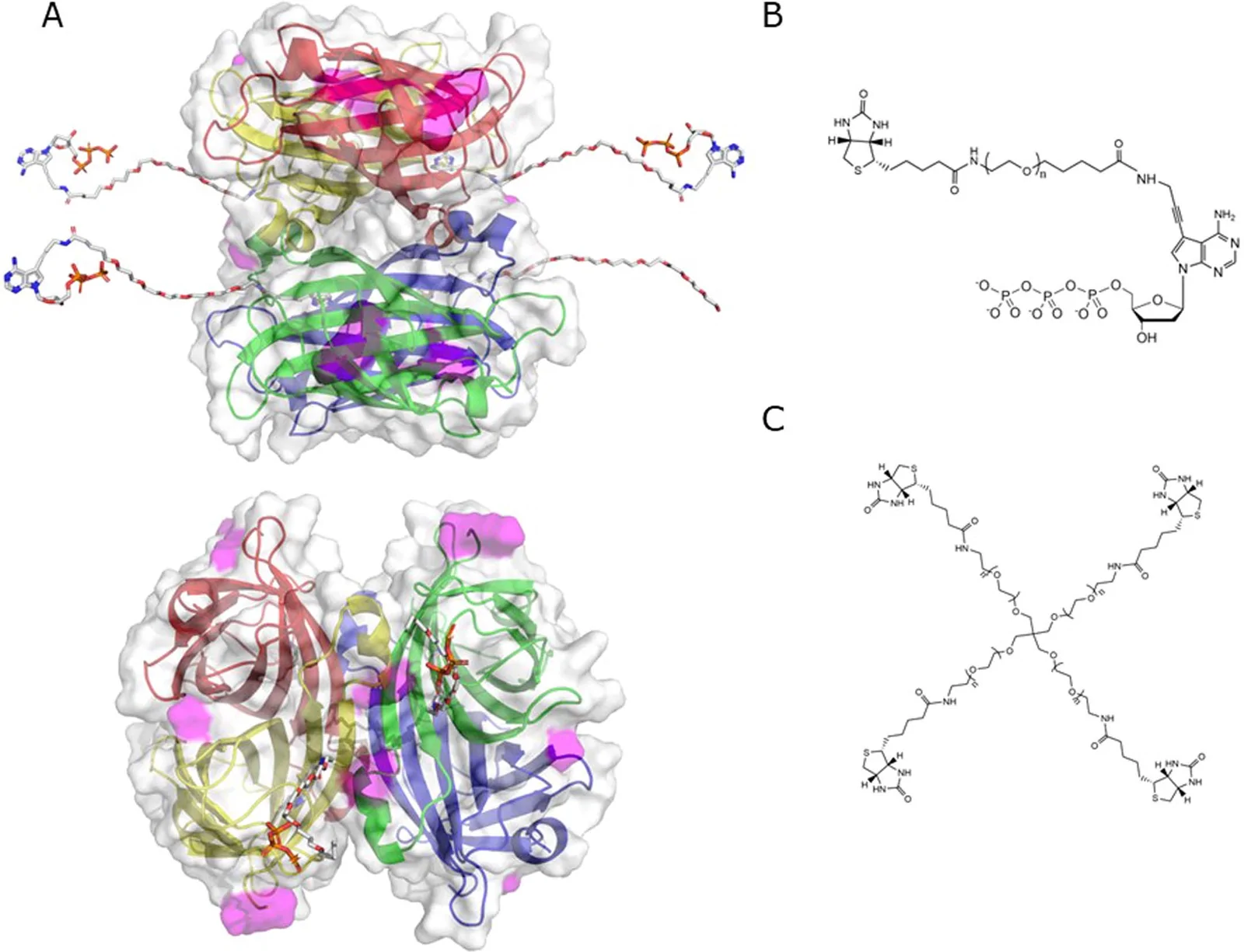
Model of an avidite. (**a**) side and top views of a modeled avidite. The protein core consists of fluorophore labeled streptavidin. The monomers of tetrameric streptavidin are colored red, blue, green, and yellow. Dye conjugation sites through lysine-NHS chemistry are denoted in the surface rendering as magenta. Fluorophores are not pictured. Avidite arms are associated via a biotin interaction with the core streptavidin protein. Arms are mixed stoichiometrically to achieve averages of three nucleotide containing arms and one linker to additional cores. Molecules conjugated to have been shortened in this representation. (**b**) Structure of an avidite arm. (**c**) Structure of the 4-arm linker connecting avidite cores.

Before sequencing, DNA fragments of interest were circularized and captured on the surface of a flowcell. Clonal copies of DNA fragments were then created through rolling circle amplification, generating approximately 1 billion concatemers on the flowcell surface^22–25^. The resulting concatemers, referred to as polonies using the original term coined by Church and collaborators^26^, were used as the DNA substrate for sequencing. In contrast to the DNA nanoballs developed by Complete Genomics, polonies are amplified on-instrument following library hybridization to the flowcell^27^. This approach simplifies user workflow and eliminates the possibility that DNA fragments may interact in solution during the amplification process. We then constructed the avidite: a dye-labeled polymer with multiple, identical nucleotides attached. In the presence of a polymerase, the avidite was able to bind multiple complementary nucleotides specifically in concatemer copies of a DNA fragment within a polony. A polymerase and a mixture of four avidites, each corresponding to a particular label and nucleotide, were applied to the flowcell and used for base discrimination. The avidite was not incorporated, but provided a stable complex while enabling removal under specifically formulated wash conditions. Removal of the avidite left no modifications in the synthesized strand. The avidites decreased the required concentration of reporting nucleotides by 100-fold relative to single-nucleotide binding, yielded negligible dissociation rates and obviated the need to have nucleotides present in the bulk solution. A low avidite concentration leads to reduced use of fluorophores relative to the strategy of using high-concentrations of dye-labeled nucleotides. The advent of the avidite enabled us to separate the process of stepping along the DNA template from the process of identifying each nucleotide, and to optimize each for quality and reagent consumption. Figure 1a shows a complete cycle of avidity sequencing, Fig. 1b depicts a single avidite interacting with multiple DNA copies within a polony and Fig. 1c shows many avidites specifically bound to several polonies on the surface. Additional detail on the structure of one version of an avidite is provided in Extended Data Fig. 1.

**Fig. 2:**
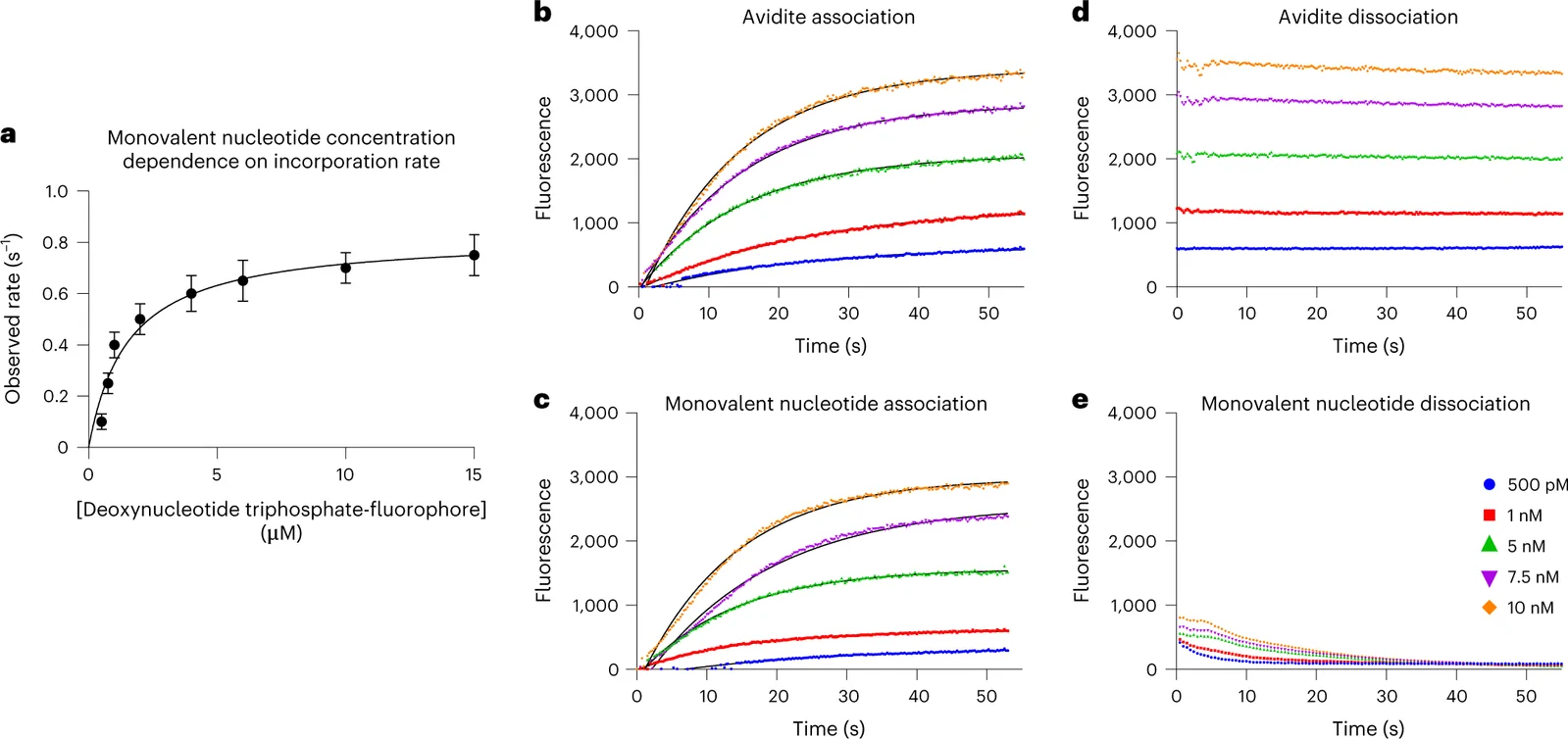
Nucleotide and avidite binding kinetics. **a**, Monovalent fluorophore-labeled nucleotide concentration dependence of the observed rate of incorporation. Time series were performed at each concentration and fit to a single exponential equation to derive a rate. Observed rates were plotted as a function of concentration and fit to a hyperbolic equation, deriving a value of *k*_pol_ = 0.86 ± 0.14 s^−1^ and *K*_d,app_ = 1.6 ± 0.6 µM. **b**,**c**, Real-time association kinetics of signal generation resulting from reacting multivalent avidite substrates (**b**) and monovalent nucleotides (**c**) with DNA polonies. **d**,**e**, Real-time measurement of signal decay following flow cell washing for imaging of multivalent avidite substrates (**d**) and monovalent nucleotides (**e**).

Avidity sequencing overcomes the kinetic challenges of generating a signal by incorporation of a dye-labeled monovalent nucleotide. In bulk solution, incorporation of a dye-labeled nucleotide is limited by a specificity constant (*k*_cat_/*K*_m_) that governs the observed rate of productive nucleotide binding and incorporation^28^. A specificity constant of 0.54 ± 0.22 µM^−1^ s^−1^ for monovalent dye-labeled nucleotides using an engineered polymerase was observed resulting from a maximum rate of incorporation (*k*_pol_) of 0.86 ± 0.14 s^−1^ and an apparent dissociation constant *K*_d_ (*K*_d,app_) of 1.6 ± 0.6 µM (Fig. 2a). This apparent *K*_d_ reflects the *K*_m_ of a kinetic system not in equilibrium rather than the true *K*_d_ of the nucleotide substrate^29^. To achieve complete product turnover, this high apparent *K*_d_ can be overcome either by using increased concentrations of fluorescent nucleotide substrate or allowing longer incorporation time for completion of the reaction. Both paths used to overcome this substrate limitation have the undesirable consequence of either high cost or long cycle time. Together, the use of avidity substrates and DNA polonies containing many copies of substrate DNA in close proximity overcomes the limitations of incorporating a monovalent dye-labeled nucleotide.

Using binding of the four labeled avidites for base identification established a binding equilibrium that reached saturation based on substrate concentration within 30 s to generate signal, rather than relying on catalysis. The binding kinetics of this interaction were monitored using real-time data collection to observe avidites binding to polonies with an association rate (*k*_on,avidite_) of 271 ± 82 nM^−1^ s^−1^ (Fig. 2b). This observed association occurred within the limit of error of a single fluorescently labeled monovalent nucleotide (Fig. 2c). Major differences were observed in the dissociation kinetics of avidite substrates versus monovalent nucleotides. Avidite substrates bound to the DNA polonies tightly with no measurable dissociation over the >1-min timescale needed for imaging and base calling (Fig. 2d). This is in sharp contrast to fluorescently labeled monovalent nucleotides, which dissociated rapidly during the wash step following binding and then continued to dissociate during imaging (Fig. 2e). The negligible dissociation rate resulted in decreased *K*_d_ of more than two orders of magnitude for avidites compared with monovalent nucleotides. With near-zero avidite dissociation rates, a persistent signal was achieved without the presence of free avidites in bulk solution, eliminating background. Without avidity, dissociation kinetics with monovalent nucleotides showed a fourfold signal decrease at the beginning of imaging due to rapid dissociation, as a result of disruption of the binding equilibrium during reagent exchange (Fig. 2e).

### Sequencing instrumentation

Avidity sequencing was performed on the AVITI commercial sequencing system. Briefly, the instrument is a four-color optical system with two excitation lines of approximately 532 and 635 nm. The four-color system is created using an objective lens, multiple tube lenses and multiple cameras for simultaneous imaging of four spectrally separated colors. The detection channels for emission are centered at approximately 553, 596, 668 and 716 nm, respectively. Reagents are delivered using a selector valve and syringe pump to perform reagent cycling. The instrument contains two fluidics modules and a shared imaging module, enabling parallel utilization of two flowcells. Subsequent to image collection, data were streamed through an onboard processing unit that performs image registration, intensity extraction and correction, base calling and quality score assignment (Methods).

### Accuracy of avidity sequencing

**Fig. 3:**
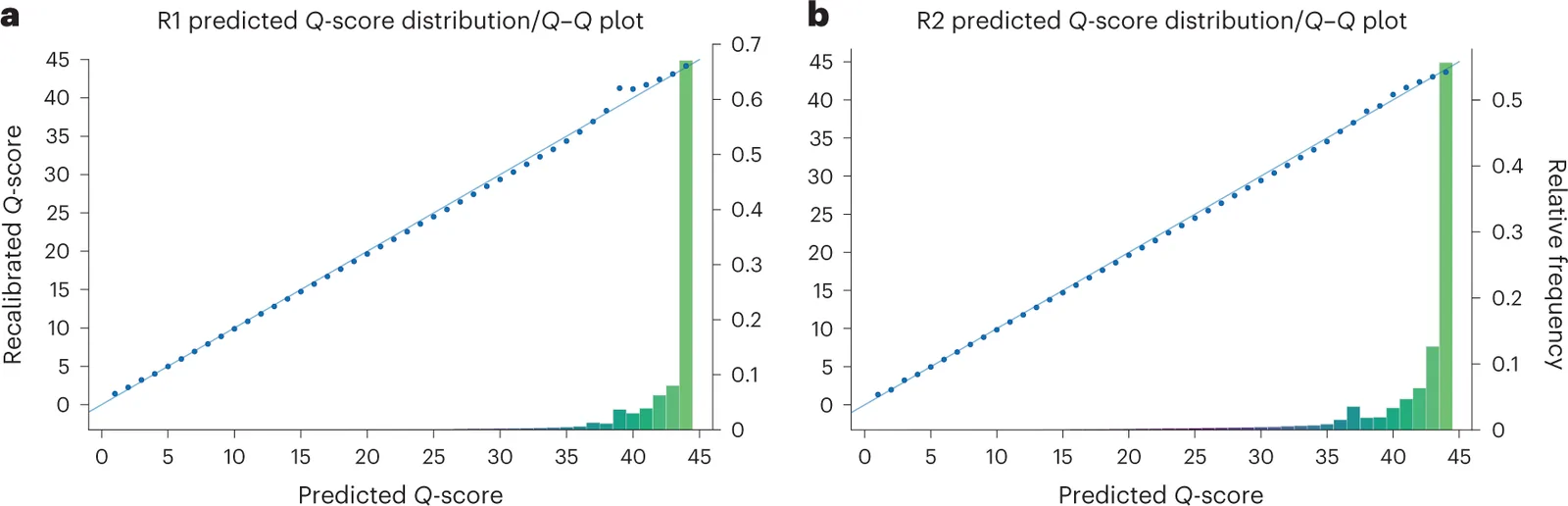
Predicted and observed quality scores for a 2 × 150-bp sequencing run of human genome HG002. **a**, Read 1 (R1). **b**, Read 2 (R2). Points on the diagonal indicate that predicted scores match observed scores. The histograms show that the majority of the data points are >*Q*40.

To evaluate the accuracy of avidity sequencing, 20 sequencing runs were performed using a well-characterized human genome. Sequencing data were used to train quality tables according to the methods of Ewing et al.^30^, but with modified predictors. Quality tables were then applied to independent sequencing runs. Figure 3 shows the data quality obtained in a representative run not used for training. Quality scores were well calibrated across the entire range, meaning that predicted quality matched observed quality as determined by alignment to a known reference. Combined over reads 1 and 2, 96.2% of base calls were >*Q*30 (an average of one error per 1,000 bp) and 85.4% >*Q*40, with a maximum of *Q*44, or approximately one error in 25,000 bases. For comparison, a publicly available PCR-free NextSeq 2000 dataset was downloaded from the Illumina public demo set repository (https://basespace.illumina.com/datacentral) and a publicly available NovaSeq 600 dataset (https://console.cloud.google.com/storage/browser/brain-genomics-public/research/sequencing/fastq). The NextSeq 2000 and NovaSeq 6000 datasets had 90.1% and 92.7% of data >*Q*30, respectively, and none of the base calls exceeded *Q*40.

**Extended Data Fig. 2:**
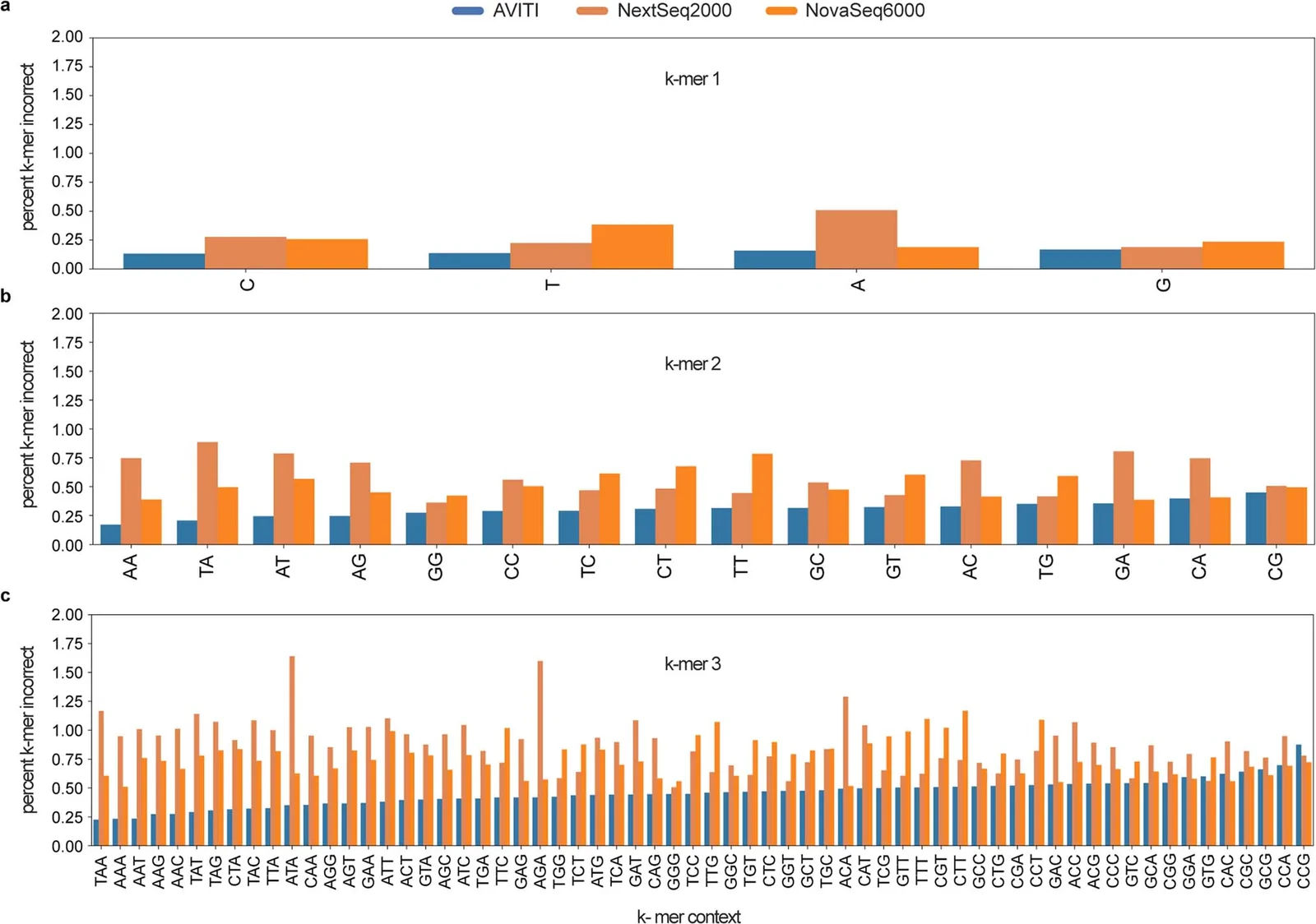
Percentage of instances that a k-mer contained at least one mismatch compared across 3 instruments. Panels **a**, **b**, and **c** display 1-mers, 2-mers, and 3-mers, respectively. The bars are sorted by AVITI contexts from most to least accurate.

To obtain an additional measure of accuracy, we used the same datasets to compute the percentage of *k*-mers (*k* = 1, 2, 3) containing at least one mismatch after alignment to a well-characterized reference. Known SNP sites were masked before the comparison. When compared with NextSeq 2000 and NovaSeq 6000, we found that AVITI had the highest accuracy across four out of four 1-mers, 16 out of 16 2-mers and 58 out of 64 3-mers (Extended Data Fig. 2).

### Homopolymer sequencing

**Fig. 4:**
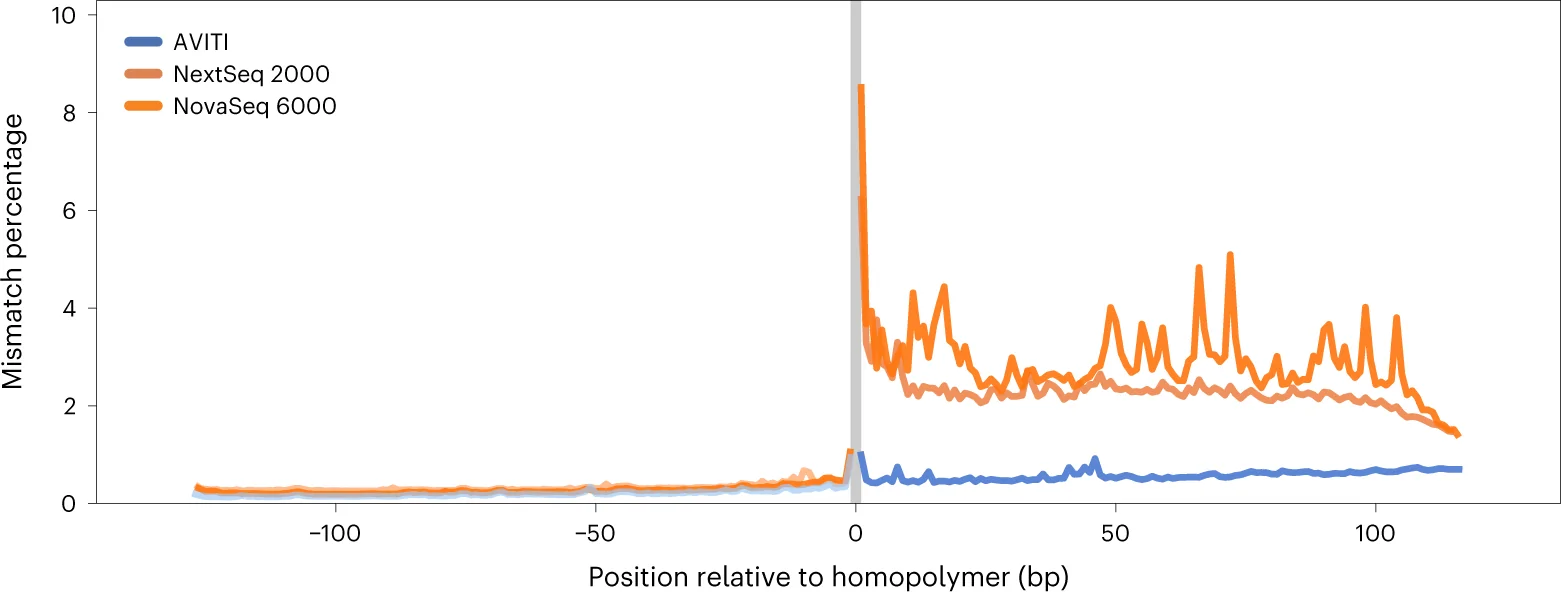
Post-homopolymer performance across platforms. Mismatch percentages of AVITI, NovaSeq 6000 and NextSeq 2000 reads before and after homopolymers of length 12 or greater.

**Fig. 5:**
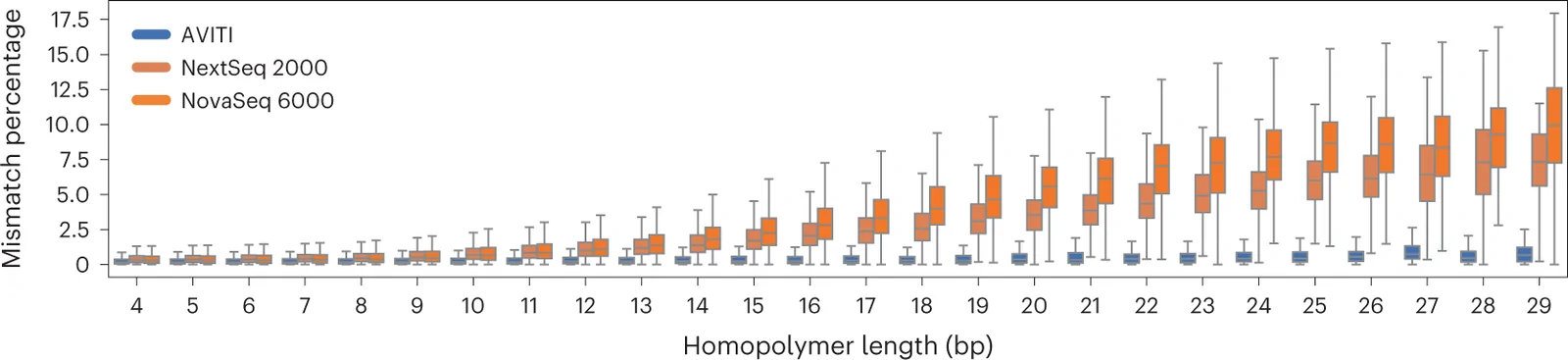
Comparison of mismatch rate following homopolymers of length between four and 29. Mismatch percentage difference between avidity sequencing and SBS increases with homopolymer length. The box plot shows median, quartiles and whiskers, which are 1.5× interquartile range.

**Extended Data Fig. 3:**
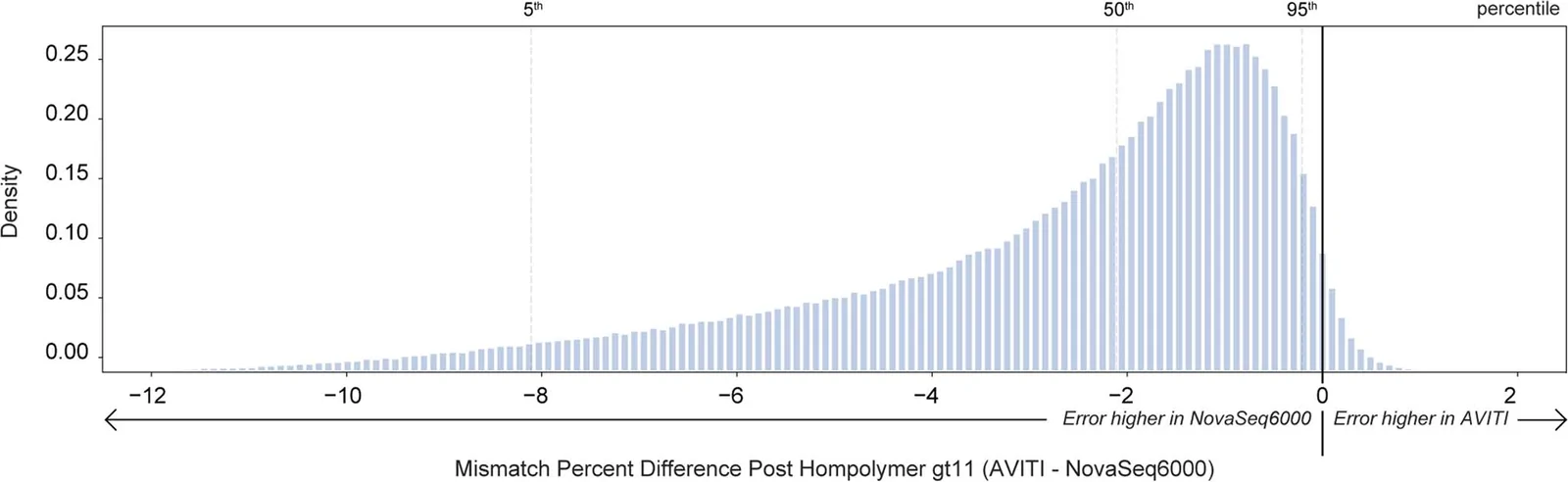
Histogram of pairwise error differences. Difference was selected as the metric to cancel the effects of human variants from the mismatch percent.

**Extended Data Fig. 4:**
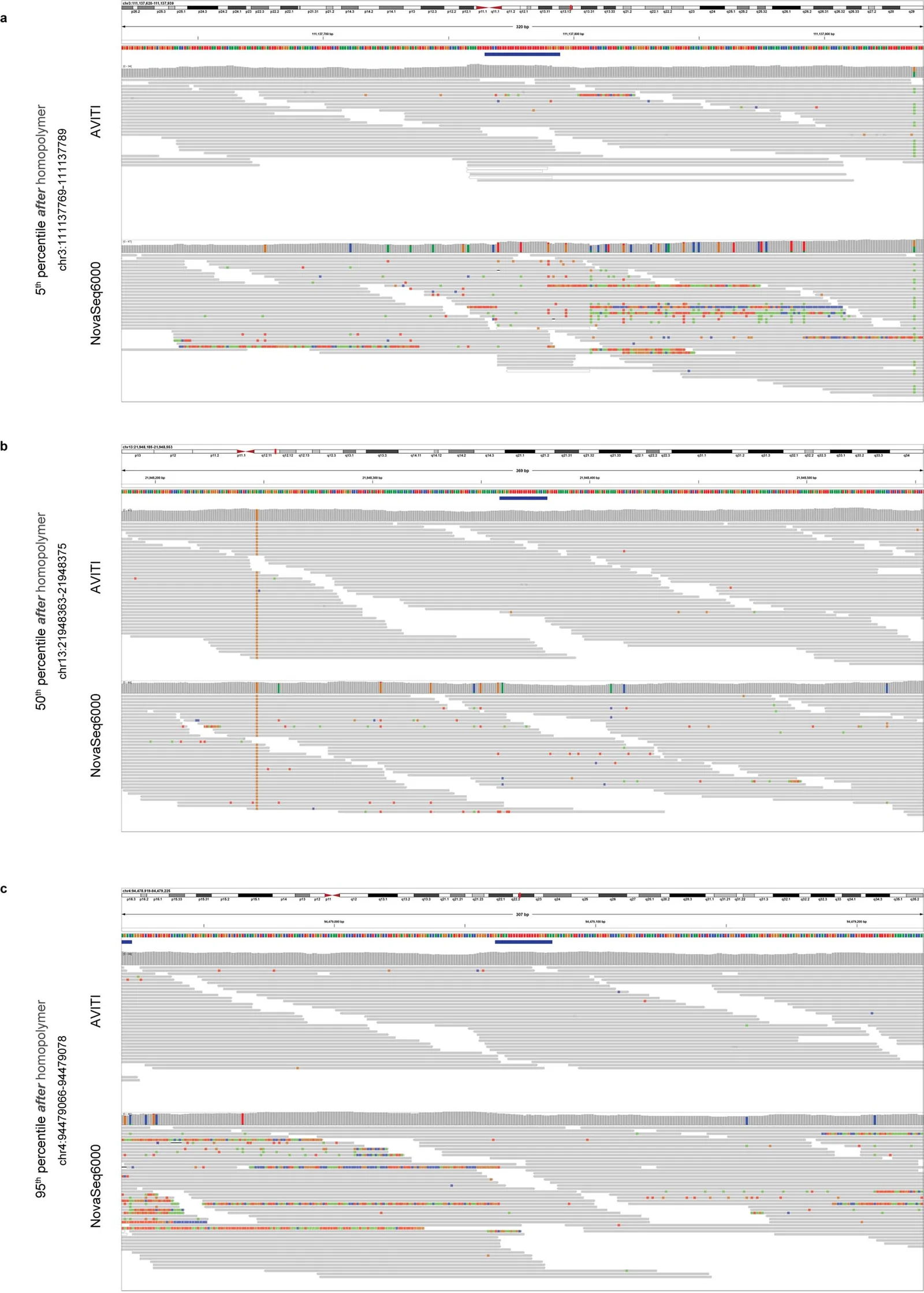
IGV display of homopolymer loci at the 5th, 50th, and 95th percentile of AVITI minus NovaSeq mismatch percent (corresponding to the dashed lines of Extended Data Fig. 3). The red bar at the top indicates the homopolymer. Colors within the IGV read stack correspond to mismatches and softclipping. Only mismatches contribute to the error rate calculation and softclipped bases are ignored.

Sequencing through long homopolymers has posed challenges for multiple sequencing technologies^31,32^. Although SBS improves homopolymer sequencing relative to flow-based technologies, the error rates of reads that pass through long homopolymer regions increase substantially^33^. Correction algorithms have been proposed to circumvent the inherent challenges with base-calling post-homopolymer repeats^34^, but the exact cause has not been fully established in the literature. In contrast to SBS, avidity sequencing leverages rolling circle amplification, polymerases evolved to accommodate the avidite complex formation and a separate polymerase evolved for efficient incorporation of unlabeled and 3′ blocked nucleotides. We evaluated the impact of these differences on sequencing through long homopolymers. Specifically, homopolymers of length 12 or more nucleotides were used to assess the accuracy of reads before and after homopolymer regions. Figure 4 shows the results comparing avidity sequencing with SBS, averaged across the ~700,000 homopolymer loci of length 12 or more. Average error rate of avidity sequencing remained stable following a long homopolymer (controlling for the fact that post-homopolymer stretch occurs in later cycles of a read). By contrast, the error rate of SBS reads increased by more than a factor of five following homopolymer stretches. Extended Data Fig. 3 shows the histogram of pairwise error rate differences between avidity sequencing and SBS for all long homopolymer loci. The avidity sequencing error rate outperformed SBS in >97% of cases and the magnitude of difference is correlated with homopolymer length (Fig. 5). Extended Data Fig. 4 shows representative loci from the 95th, 50th and fifth percentiles of the histogram.

### Single-cell RNA-seq

To demonstrate sequencing performance across common applications, single-cell RNA expression libraries were prepared and sequenced. Two libraries from a reference standard consisting of human peripheral blood mononuclear cells were generated using the 10X Chromium instrument. The two libraries contain RNA from roughly 10,000 and 1,000 cells, respectively. Following circularization, the libraries were sequenced to generate paired-end reads with read lengths of 28 and 90 for reads 1 and 2, respectively, as recommended by the vendor. The analysis was done using CellRanger (https://support.10xgenomics.com/single-cell-gene-expression/software/pipelines/latest/installation). Because this reference standard is used by 10X Genomics to evaluate sequencing performance, a set of metrics and guidelines to assess sequencing results is provided along with the biological material. Extended Data Table 1 shows each metric, the guideline values from 10X Genomics and the performance of each sequenced library. All metrics were within the guide ranges, and metrics pertaining to sequencing quality exceeded the thresholds provided.

### Whole-human-genome sequencing

**Extended Data Fig. 5:**
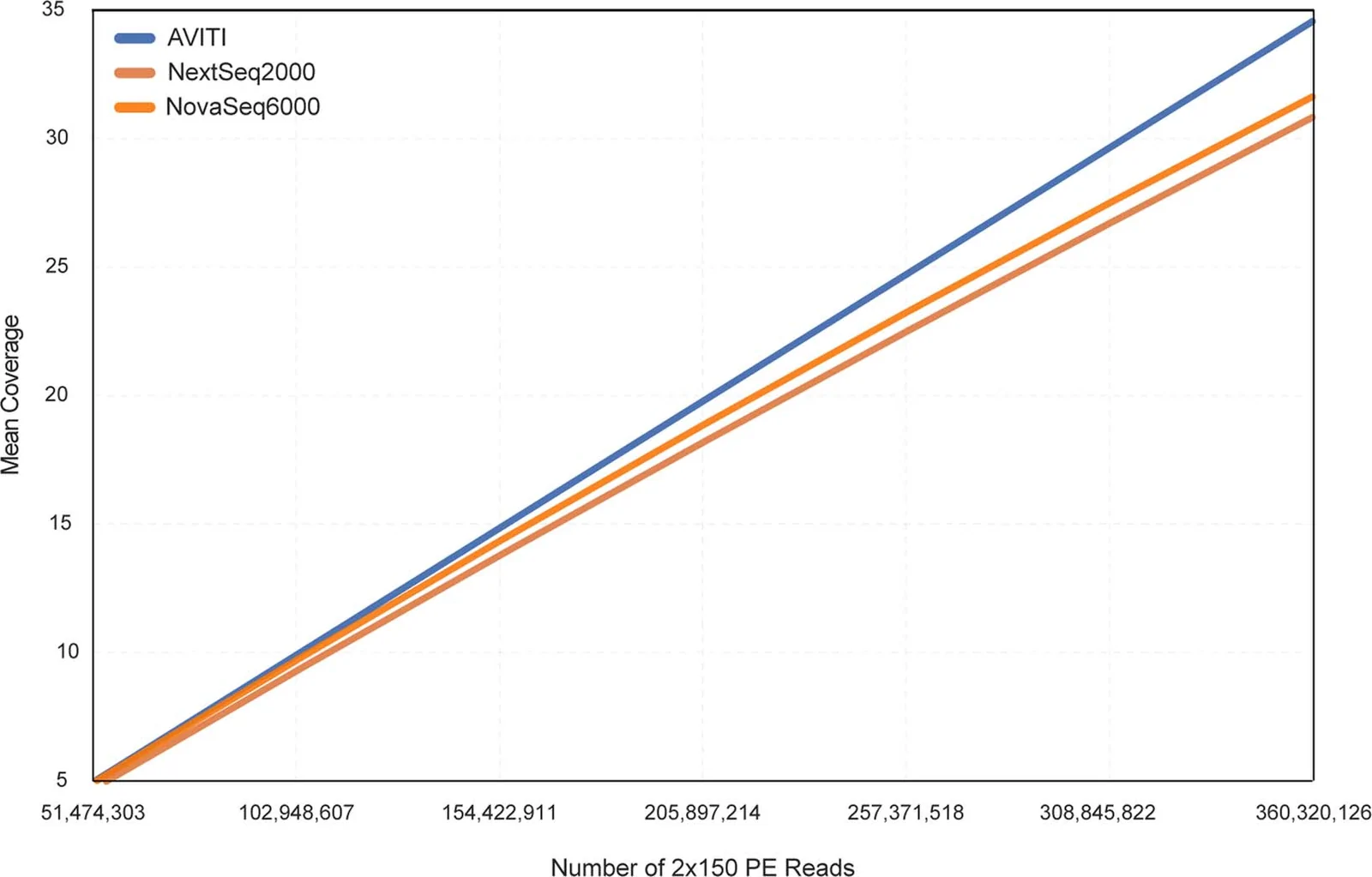
Comparison of read number vs genomic coverage computed via Picard for PCR-free whole genome data. AVITI most closely matches the 45-degree line due to the low duplicate rate.

Another common application is human-whole-genome sequencing. This application challenges sequencer accuracy to a greater extent than measurement of gene expression because the latter requires only accurate alignment while the former depends on nucleotide accuracy to resolve variant calls. To demonstrate performance for this application, the well-characterized human sample HG002 was prepared for sequencing using a Covaris shearing and PCR-free library preparation method and sequenced with 2 × 150-bp reads. The run generated 1.02 billion passing filter paired-end reads with a duplicate rate of 0.58% (0.11% classified as optical duplicates by Picard (https://broadinstitute.github.io/picard/)). To underscore the impact of low duplicates, we compared the number of input reads with genomic coverage (Extended Data Fig. 5).

**Extended Data Fig. 6:**
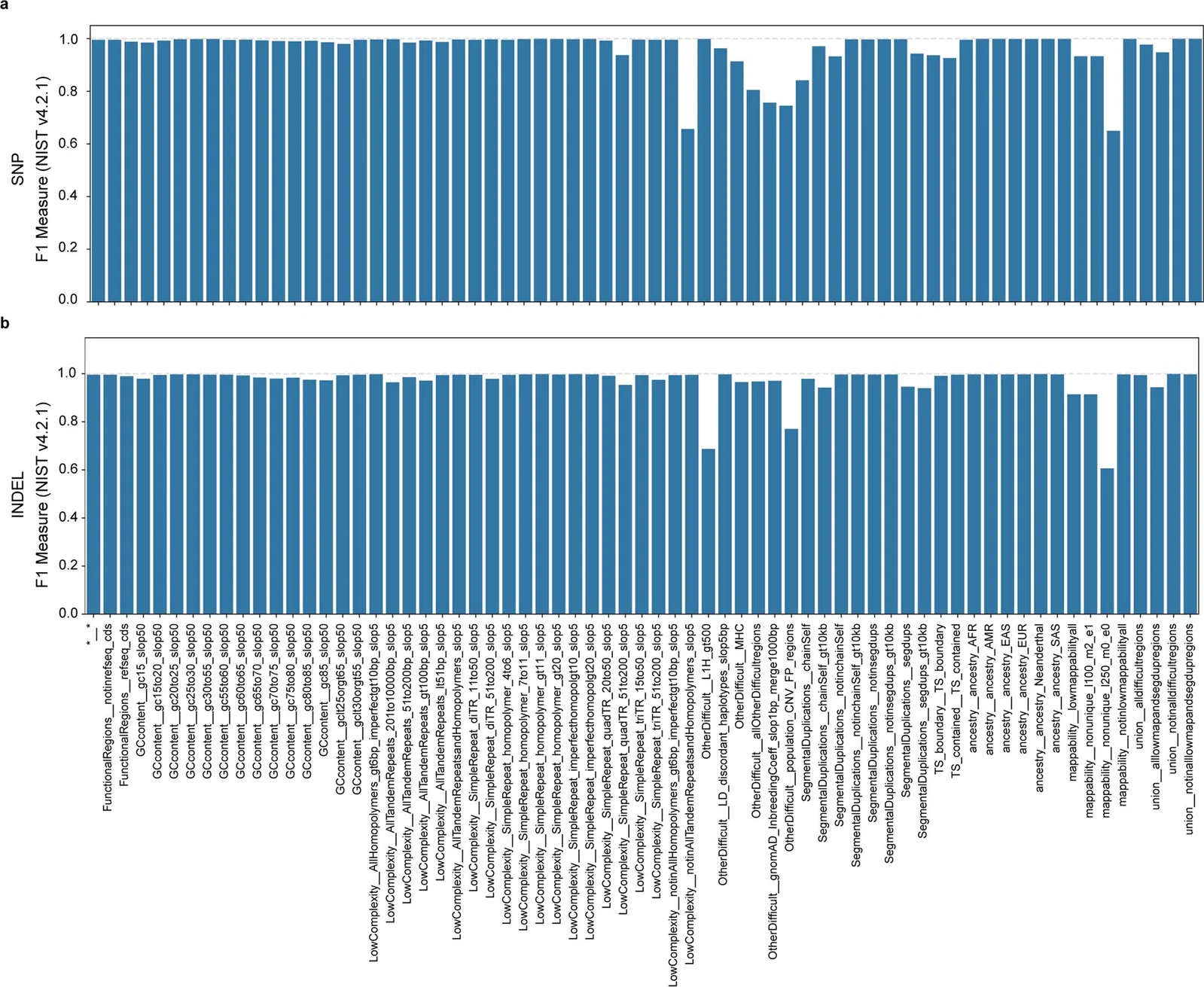
F1 Score of SNPs and indels across GiaB stratifications. F1 score for SNPs and indels stratified by all GiaB regions with at least 100 variants in the 4.2.1 truth set of sample HG002.

A FASTQ file with the base calls and quality scores was downsampled to 35-fold coverage and used as an input into the DNAScope analysis pipeline from Sentieon. SNP and indel calls achieved F1 scores of 0.995 and 0.996, respectively. Extended Data Table 2 shows variant-calling performance for SNPs and small indels on the GIAB-HC regions. Sensitivity, precision and F1 scores are shown. The performance on SNPs and indels is comparable. Extended Data Fig. 6 shows the F1 score for SNPs and indels across all GiaB stratifications with at least 100 variants in the truth set.

### Extensibility of avidity sequencing

**Fig. 6:**
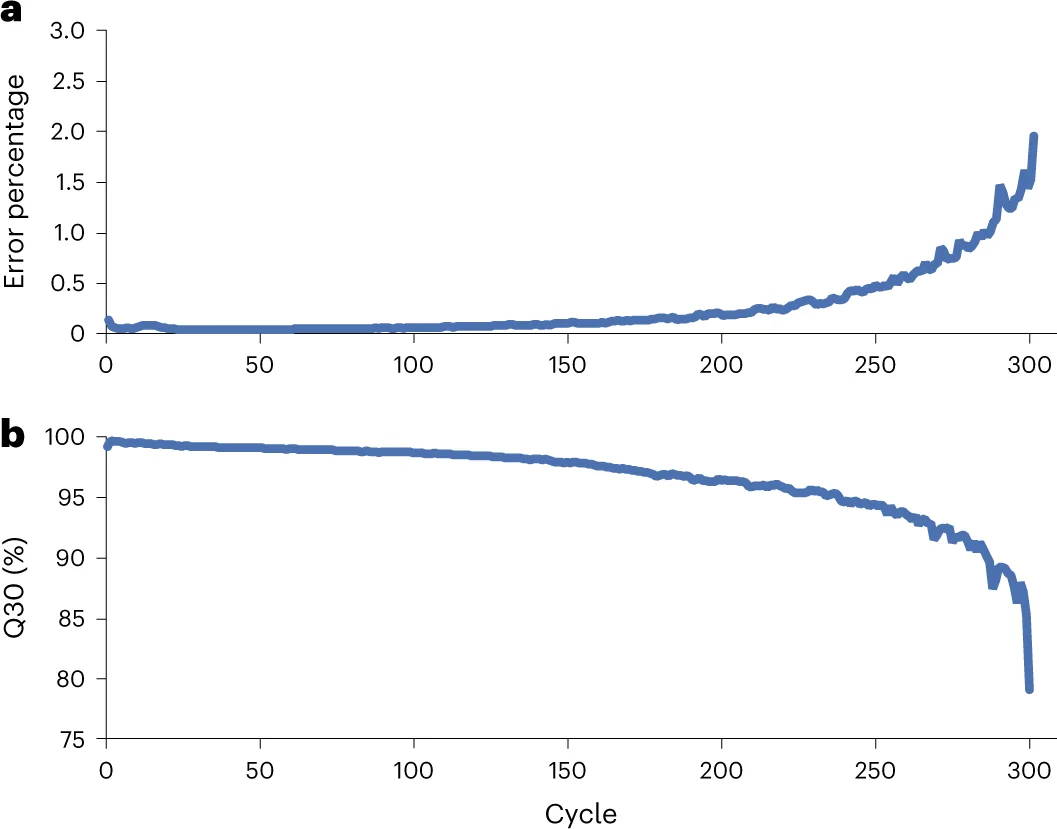
Performance of a 300-cycle *E. coli* sequencing run. **a**, Percentage *Q*30 by cycle. Overall *Q*30 percentage exceeds 96% and end of read has 85% *Q*30. **b**, *E. coli* error rate as a function of cycle. Alignment settings strongly discourage soft clipping, and >99% of reads pass filter. Final cycle error rate was 0.019.

To assess the extensibility of avidity chemistry we continued a sequencing run beyond 150 bp to generate a 1 × 300 dataset from an *Escherichia coli* library. To achieve this we used both an optimized polymerase and an optimized reagent formulation. Figure 6a shows quality scores as a function of sequencing cycle. Because quality scores were not trained to these lengths, the scores are approximate. Figure 6b shows the *E. coli* error rate as a function of cycle number based on alignment to the known reference strain. The error rate of the final cycle was 1.9% and that at cycle 150 was 0.1%. Error calculations were based on the vast majority of the data with a pass filter rate for the run of >99.6% and Burrows–Wheeler aligner (BWA) settings aimed at strongly discouraging soft clipping (no cycles with soft clipping >0.04%). The enzymes and formulations developed for this run will be leveraged as we continue to identify extensions and improvements.

## Discussion

We present a sequencing chemistry that achieves improved quality and lower reagent consumption by independent optimization of nucleotide incorporation and signal generation. Although other chemistries have proposed the separation of incorporation and signal generation^35^, the avidite concept benefits from the fact that multiple nucleotides on the avidite bind multiple copies of the DNA template within a polony, which decreases dissociation rate constant and the labeled reagent concentration requirement for base classification. Furthermore, the avidite construct is modular. The core can be swapped for a different substrate. Both number and type of dye molecules are configurable, and many types of linkers can be used. The changes are straightforward to implement and do not require modification of the polymerase responsible for binding the nucleotides attached to the linkers. The modular design speeds technology improvement because each component can be optimized in parallel for increased signal, decreased cycle time, lower reagent concentration or any other potential axis of improvement.

The avidity chemistry described above has been implemented as part of a benchtop sequencing solution. The accuracy of the sequencer was demonstrated by training a quality model on human sequencing data, which shows that in the majority of bases in an independent human-whole-genome sequencing run is >*Q*40. The high level of accuracy probably results from (1) the use of an engineered high-fidelity polymerase, (2) synergistic binding of multiple nucleotides on a single avidite to ensure only the correct cognate avidite binds to the polony and (3) a binding disadvantage for out-of-phase DNA copies within a polony that lack other out-of-phase neighbors to serve as avidity substrates. Future work will be required to investigate the relative contribution of each mechanism proposed above. In addition to overall accuracy improvements, the chemistry retains good performance in reads containing long homopolymers. The sequencer can be used in a wide range of applications, as exemplified by results for single-cell RNA-seq and for whole-human-genome sequencing. In both cases, reference standards were sequenced so that the quality of result could be assessed. The single-cell data exceeded the quality metric guidelines provided by 10X Genomics (https://www.10xgenomics.com/compatible-products?query=&page=1). The human genome variant-calling results showed high sensitivity and precision for both SNPs and small indels^36^. The two benchmarking studies were selected due to the availability of well-characterized samples and because they represent very different use cases. However, these are only examples and other applications have been demonstrated, including whole-genome sequencing for rare disease^37^, low-pass sequencing with imputation^38^ and single-cell sequencing of DNA and RNA^39^. Although the current implementation of avidity-based sequencing already achieves high accuracy and broad applicability, there are many improvement directions being explored. In addition to the initial demonstration of longer reads shown here, further quality improvements, shorter cycle times and higher densities are under development.

## Methods

### Solution measurements of nucleotide incorporation

Solution measurements of nucleotide kinetics were performed using commercially available dATP-Cy5 (Jena Bioscience, catalog no. NU-1611-CY5-S). DNA substrates for solution kinetic assays were prepared by annealing a 5′FAM-labeled primer oligo (purchased from IDT) and high-performance liquid chromatography-purified (5′-CGAGCCGTCCAACCTACTCA-3′) with a template oligo (5′-ACGACCATGTTGAGTAGGTTGGACGGCTCG-3′). Annealing was performed with 10% excess template oligo in the annealing buffer using a PCR machine to heat oligos to 95 °C, followed by slow cooling to room temperature over 60 min. Solution kinetics were performed by mixing a preformed enzyme–DNA complex with fluorescent nucleotide and MgSO_4_ using a RQF3 Rapid Quench Flow (KinTek Corp.). The enzyme used was an engineered variant of *Candidatus altiarchaeales* archaeon. The final reaction was conducted in 25 mM Tris pH 8.5, 40 mM NaCl and 10 mM ammonium chloride at 37 °C. Extension products were separated from unextended primer oligos by capillary electrophoresis using a 3500 Series Genetic Analyzer (ThermoFisher) to achieve single-base resolution. Products were quantified and fit to a single exponential equation. The observed rates as a function of nucleotide concentration were then fit to a hyperbolic equation to derive apparent *K*_d_ (*K*_d,app_) and rate of polymerization (*k*_pol_).

### Avidite synthesis and construction

Initial research scale avidites were constructed by dissolving 5 mg of 10 kD 4-arm-PEG-SG (Laysan Bio, catalog no. 4arm-PEG-SG-10K-5g) in 100 µl of 95% organic solvent (for example, ethanol) and 5 mM MOPS pH 8.0 to make a 50 mg ml^–1^ solution (5 mM), 19 µl of which was combined with 1.5 µl of 10 mM dATP-NH_2_ (7-deaza-7-propargylamin′-2′-deoxyadenosin′-5′-triphosphate; Trilink, catalog no. N-2068) and 8.0 µl of 3.75 mM 2 kD Biotin-PEG-NH_2_ (Laysan Bio, catalog no. Biotin-PEG-NH2-2K-1g) in 95% organic solvent (for example, ethanol) and 5 mM MOPS pH 8.0. After mixing, 5 mM 10 kD 4-arm-PEG-SG was added. The final composition was 0.50 mM dA-NH_2_, 1.0 mM biotin-PEG-NH2 (2 kD), 0.25 mM 4-arm-PEG-NHS, 85.5% organic solvent (for example, ethanol) and 4.5 mM MOPS pH 8.0. Following 1,000-rpm incubation at 25 °C for 90 min, the reaction volume was adjusted to 100 µl by the addition of MOPS pH 8.0. Purification was performed using a Biorad Biospin P6 column pre-equilibrated in 10 mM MOPS pH 8.0. The purified dATP-PEG–biotin complex was mixed with Zymax Cy5 Streptavidin (Fisher Scientific, catalog no. 438316) in a 2.5:1 volumetric ratio and allowed to equilibrate for 30 min at room temperature.

### Real-time measurement of avidite association and dissociation

Real-time measurement of avidite binding kinetics was performed using an Olympus IX83 microscope at 545 and 635 nm excitation (Lumencor Light Engine) set to an approximate power density of about 1 W cm^–2^, with an Olympus objective (catalog no. UCPLFLN20XPH) and a Semrock BrightLine multiband laser filter set (catalog no. LF405/488/532/635) containing a matching quad band exciter, emitter and dichroic. Flow rates of 60 µl s^–1^ were used for reagent exchanges. Circular PhiX libraries were introduced to AVITI flow cells, hybridized in 3× SSC buffer for 5 min at 50 °C and cooled to room temperature. Amplification reagents were introduced into the flow cell to perform rolling circle amplification and amplify genomic DNA. The instrument was paused following polony generation and priming and the flowcell moved to the microscope. Custom control software was written to control all peripheral hardware and synchronize data collection with flow of materials into the sample. Data collection (4 fps) was triggered by flow of the avidity mix and collected for 55 s. Polonies in the field were localized by a spot-finding algorithm, and background-corrected intensities were extracted versus time. Experiments were performed at 0.5 pM, 1 nM, 7.5 nM and 10 nM avidite or monovalent dye-labeled nucleotide concentrations. Substrates at the respective concentrations were combined with 100 nM engineered enzyme variant of *C. altiarchaeales* archaeon in the avidity on rate assay buffer formulation (25 mM HEPES pH 8.8, 25 mM NaCl, 0.5 mM EDTA, 5 mM strontium acetate, 25 mM ascorbic acid and 0.2% Tween-20). Avidites and nucleotides were labeled with Alexa Fluor 647. Higher-concentration data collection was limited by the ability to detect polony intensity from free avidite intensity at elevated concentrations. Off-rate measurements were performed by binding avidites to flowcell polonies, followed by washing with avidity on rate assay buffer and triggering of data collection.

### Genomic DNA and next-generation sequencing library preparation

Human DNA from cell line sample HG002 was obtained from the Coriell Institute. Linear next-generation sequencing library construction was performed using a KAPA HyperPrep library kit (Roche, catalog no. 07962363001) according to published protocols. Finished linear libraries were circularized using the Element Adept Compatibility kit (catalog no. 830-00003). Final circular libraries were quantified by quantitative PCR with the standard and primer set provided in the kit. Circular library DNA was denatured using sodium hydroxide and neutralized with excess Tris pH 7.0 before dilution. Denatured libraries were diluted to 8 pM in hybridization buffer before loading onto the sequencing cartridge.

### Single-cell 3′ gene expression library circularization

Single-cell RNA-seq libraries were prepared from two lots of peripheral blood mononuclear cell suspension (10,000 and 1,000 cells) using the Chromium Next GEM Single Cell 3′ Kit v.3.1 (catalog no. 1000268). Each library was quantified and individually processed for sequencing using the Adept Library Compatibility Kit (catalog no. 830-00003). Processed libraries were pooled and sequenced with 28 cycles for read 1, 90 for read 2 and index reads.

### Sequencing instrument and workflow

Sequencing results were obtained with commercialized formulations of avidites, enzymes and buffers. Element Bioscience’s AVITI commercial system (catalog no. 88-00001) was used for all sequencing data. AVITI 2 × 150 kits were loaded on the instrument (catalog no. 86-00001). Primary analysis was performed onboard the AVITI sequencing instrument, and FASTQ files were subsequently analyzed using a secondary analysis pipeline from Sentieon.

### Sequencing primary analysis

Four images were generated per field of view during each sequencing cycle, corresponding to the dyes used to label each avidite. An analysis pipeline was developed that uses the images as input to identify the polonies present on the flowcell and to assign to each polony a base call and quality score for each cycle, representing the accuracy of the underlying call. The analysis approach has steps similar to those described in ref. ^25^. Briefly, intensity is extracted for each polony in each color channel; intensities are then corrected for color cross-talk and phasing and normalized to make cross-channel comparisons. The highest normalized intensity value for each polony in each cycle determines the base call. In addition to assigning a base call, a quality score corresponding to call confidences is also assigned. The standard *Q*-score definition is utilized where the *Q*-value is defined as *Q* = −10 × log_10*p*, where *p* is the probability that the base call is an error. *Q*-score generation follows the approach of Ewing et al., with modified predictors^21^, and is encoded using the phred+33 ASCII scheme. The predictors used for quality score training are (1) maximum intensity per polony across color channels; (2) clarity of each polony (defined as (*A* + 1)/(*B* + 1), where *A* is the highest intensity across color channels and *B* is the second highest); (3) the sum of phasing and prephasing estimates; and (4) the median clarity value taken across the 10% of the lowest-intensity polonies. The sequence of base call assignments and quality scores across the cycles constitutes the output of the run. These data are represented in standard FASTQ format for compatibility with downstream tools.

### Quality score assessment

To assess the accuracy of quality scores (Fig. 3), the FASTQ files were aligned with BWA to generate BAM files. GATK BaseRecalibrartor was then applied to the BAM, specifying files of publicly available known sites to exclude human variant positions.

### *K*-mer error analysis

The same run used to generate recalibrated quality scores was analyzed via custom script for all *k*-mers of size 1, 2 and 3. The computation is based on 1% of a 35X genome to ensure adequate sampling of each *k*-mer. For example, each 3-mer is sampled at least 850,000 times (average 6.7 million). This figure is based on a publicly available run from each platform. For the instances of each *k*-mer, the percentage mismatching a variant-masked reference was computed. The same script was applied to a publicly available NovaSeq dataset for HG002 and a publicly available NextSeq 2000 dataset for HG001 (Demo Data for HG002 were not available). We tabulated the number of *k*-mers in which the percentage incorrect was lowest for AVITI among the three platforms compared.

### Homopolymer analysis

A BED file provided by National Institute of Standards and Technology (NIST) genome-stratifications v.3.0, containing 673,650 homopolymers of length >11, was used to define regions of interest for homopolymer analysis (GRCh38_SimpleRepeat_homopolymer_gt11_slop5). Reads overlapping these BED intervals (using samtools view -L and adjusting for slop5) were selected for accuracy analysis. Reads with any of the following flags set were discarded: secondary, supplementary, unmapped or reads with mapping quality of 0. Reads were oriented in the 5′→3′ direction and split into three segments: preceding the homopolymer, overlapping it and following it. The mismatch rate for each read segment was computed, excluding N-calls, softclipped bases and indels. For example, if a 150-bp read (aligned on the forward strand) contained a homopolymer in positions 100–120, the first 99 cycles were used to compute the error rate before the homopolymer and the last 30 to compute error rate following the homopolymer. Reads were discarded if the sequence either preceding or following the homopolymer was <5 bp in length. All reads were then stacked into a matrix according to their positional offset relative to the homopolymer, and error rate per post-offset was computed.

Average error rate was computed for avidity sequencing runs and for publicly available data from multiple SBS instruments, for comparison. Differences oin mismatch percentage, across all BED intervals, between AVITI and NovaSeq were plotted in a histogram and examples showing various percentiles within the distribution were chosen for display via Integrative Genomics Viewer.

Publicly available datasets for NovaSeq were obtained from the Google Brain Public Data repository on Google Cloud (https://console.cloud.google.com/storage/browser/brain-genomics-public/research/sequencing/fastq). Publicly available NextSeq 2000 data were obtained from Illumina Demo Data on BaseSpace (https://basespace.illumina.com/datacentral).

### Single-cell gene expression data analysis

Following sequencing, Bases2Fastq software was used to generate FASTQ files for compatible upload into 10X Cloud and subsequent analysis with the 10X Genomics Cell Ranger analysis package. Data visualization of single-cell gene expression profiling was generated using 10X Genomics Loupe Browser.

### Whole-genome sequencing analysis

A FASTQ file with base calls and quality scores was downsampled to 35× raw coverage (360,320,126 input reads) and used as an input into Sentieon BWA followed by Sentieon DNAscope^40^. Following alignment and variant calling, variant calls were compared with the NIST genome in Bottle Truth Set v.4.2.1 via the hap.py comparison framework to derive total error counts and F1 scores^41^. The results are computed based on the 3,848,590 SNV and 982,234 indel passing variant calls made by DNAScope.

### 1 × 300 Data generation

An *E. coli* library was prepared using enzymatic shearing and PCR amplification. The library was then sequenced for 300 cycles using new enzymes for stepping along the DNA template and for avidite binding. The reagent formulation with increased enzyme and nucleotide concentrations during the stepping process was used to improve stepping performance. The contact times for avidite binding and exposure were both reduced without performance losses, to decrease cycle time over the 600 cycles of sequencing. The displays show only 299 cycles of data, because cycle 300 was used only for prephasing correction. To minimize soft clipping during alignment the following inputs were used in the call to BWA–MEM: -E 6,6 -L 1000000 -S.

### Reporting summary

Further information on research design is available in the Nature Portfolio Reporting Summary linked to this article.

## Online content

Any methods, additional references, Nature Portfolio reporting summaries, source data, extended data, supplementary information, acknowledgements, peer review information; details of author contributions and competing interests; and statements of data and code availability are available at https://doi.org/10.1038/s41587-023-01750-7.

## Supplementary information


Reporting Summary


## Figures and Tables

**Extended Data Table 1:**
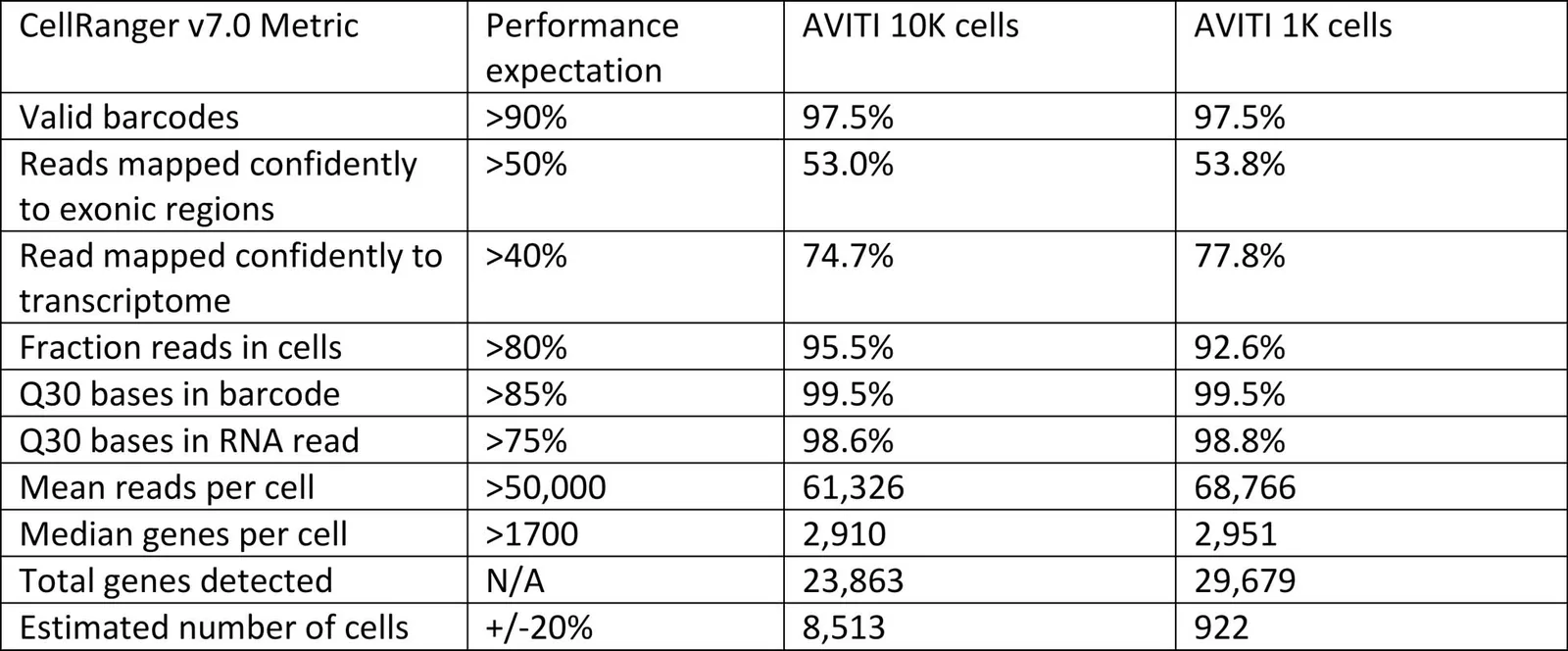
Single cell expression: CellRanger metric values for 10 K cell and 1 K cell libraries from the PBMC reference

**Extended Data Table 2:**
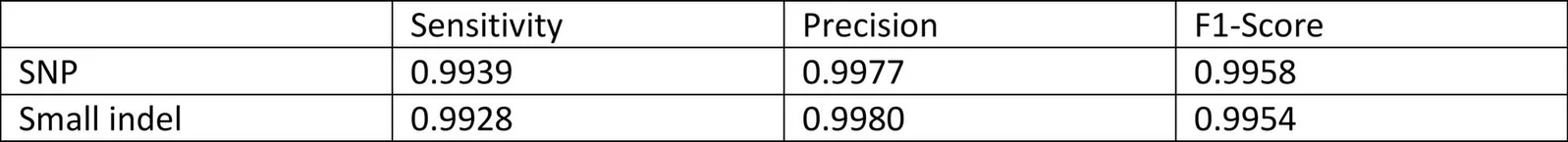
Variant calling performance for HG002 on GIAB-HC regions

## Data Availability

The avidity sequencing datasets described in the paper are available for download via the AWS CLI in the public bucket s3://avidity-manuscript-data/, pending upload to the sequence read archive under BioProject PRJNA869673. Publicly available datasets for NovaSeq were obtained from the Google Brain Public Data repository on Google Cloud (https://console.cloud.google.com/storage/browser/brain-genomics-public/research/sequencing/fastq). Publicly available NextSeq 2000 data were obtained from Illumina Demo Data on BaseSpace (https://basespace.illumina.com/datacentral).

## Code availability

Scripts used for analysis are available via GitHub (https://github.com/Elembio/AvidityManuscript2023).
