# Supplementary material for: Sequencing by avidity enables high accuracy with low reagent consumption
Source: Nat Biotechnol. 2023 May 25;42(1):132–8. doi: 10.1038/s41587-023-01750-7 (PMC10791576; doi:10.1038/s41587-023-01750-7)
Supplement: Supplementary file 1 — Reporting Summary [file 41587_2023_1750_MOESM1_ESM.pdf]

nature portfolio

Corresponding author(s): Michael Previte

Last updated by author(s): 3/7/2023

Reporting Summary

Nature Portfolio wishes to improve the reproducibility of the work that we publish. This form provides structure for consistency and transparency in reporting. For further information on Nature Portfolio policies, see our Editorial Policies and the Editorial Policy Checklist.

Statistics

For all statistical analyses, confirm that the following items are present in the figure legend, table legend, main text, or Methods section.

n/a	Confirmed	
☐	☒	The exact sample size (*n*) for each experimental group/condition, given as a discrete number and unit of measurement
☐	☒	A statement on whether measurements were taken from distinct samples or whether the same sample was measured repeatedly
☒	☐	The statistical test(s) used AND whether they are one- or two-sided<br>*Only common tests should be described solely by name; describe more complex techniques in the Methods section.*
☒	☐	A description of all covariates tested
☐	☒	A description of any assumptions or corrections, such as tests of normality and adjustment for multiple comparisons
☐	☒	A full description of the statistical parameters including central tendency (e.g. means) or other basic estimates (e.g. regression coefficient) AND variation (e.g. standard deviation) or associated estimates of uncertainty (e.g. confidence intervals)
☒	☐	For null hypothesis testing, the test statistic (e.g. *F*, *t*, *r*) with confidence intervals, effect sizes, degrees of freedom and *P* value noted<br>*Give P values as exact values whenever suitable.*
☒	☐	For Bayesian analysis, information on the choice of priors and Markov chain Monte Carlo settings
☒	☐	For hierarchical and complex designs, identification of the appropriate level for tests and full reporting of outcomes
☒	☐	Estimates of effect sizes (e.g. Cohen's *d*, Pearson's *r*), indicating how they were calculated

Our web collection on statistics for biologists contains articles on many of the points above.

Software and code

Policy information about availability of computer code

Data collection	Kinetic data for Figure 2A was collected using RQF3 Rapid Quench flow (Kintek corporation). Real-time measurements for Figure 2B-E were collected on an Olympus IX83 microscope equipped with 545 and 637 lines (Lumencor), Semrock brightline multiband laser filter set (LF405/488/532/635) containing matching quad band exciter, emitter and dichroic. Flow was induced by a syringe pump pulling reagents across an AVITI flow cell at a rate of 60 ul/s. Prior to injection of reagents, real-time data was collected on an Andor sCMOS camera at 4 frames/s. All sequencing data was collected on the AVITI commercial instrument.
Data analysis	Kinetic data was analyzed and fit using conventional non-linear regression. All error bounds were propagated in the analysis and are reflected in figure 2 panel A. Reported kcat and Kd,app were obtained by fitting to a hyperbolic equation using no constrains other than the error reported for each point.<br>Primary analysis of the collected data was performed on the AVITI instrument according to similar steps described on Whiteford et al. (25) FASTQ were generated using the bases2fastq software toolkit (version 1.1.1).<br>Tools and scripts supporting bioinformatic analysis of this manuscript can be found at the following repo located on github - https://github.com/Elembio/AvidityManuscript2023.<br>Single cell RNA was performed using CellRanger (version 7.0.1).<br>Whole genome sequencing analysis was performed by first down-sampling the input FASTQ to 35X raw coverage (360,320,126, 2x150 input reads), and then aligning, de-duplicating and sorting using sentieon bwa (version 202112.02). The BAM was then used as input to Sentieon DNAscope (version 202112.02) in addition to a element specific ML model (SentieonDNAscopeModelElementBio0.3.model) to produce a VCF. Following alignment and variant calling, the variant calls were benchmarked using hap.py (version hap.py-0.3.14) to the NIST genome in a bottle truth set v4.2.1 across all regions to derive total error counts and F1 scores.<br>To assess the accuracy of quality scores shown in Fig. 3, the aligned BAMS were processed using GATK BaseRecalibrartor (version gatk4:4.2.0.0—0), and specifying publicly available known sites files to exclude human variant positions (HG002 NIST v4.2.1 bed/vcf,

1000G_phase1.snps.high_confidence.hg38, dbsnp_144.hg38). The resulting predicted and recalibrated q-scores were plotted.
To compute the mismatch percentage of AVITI, NovaSeq 6000, and NextSeq 2000 reads before and after homopolymers of length 12 or greater, a BED file provided by NIST genome-stratifications v3.0, containing 673,650 homopolymers of length greater than 11 was used to define the regions of interest for the homopolymer analysis (GRCh38_SimpleRepeat_homopolymer_gt11_slop5). Reads that overlapped these BED intervals (using samtools version 1.1.1) were selected for accuracy analysis. Reads with any of the following flags set were discarded (secondary, supplementary, unmapped or reads with mapping quality of 0). Reads were oriented in the 5′ -> 3′ direction, and split into 3 segments, preceding the homopolymer, overlapping the homopolymer, and following the homopolymer. The mismatch rate for each read-segment was computed, excluding N-calls, softclipped bases and indels. For example, if a 150 bp read (aligned on the forward strand) contains a homopolymer in positions 100-120, then the first 99 cycles were used to compute the error rate prior to the homopolymer, and the last 30 cycles were used to compute the error rate following the homopolymer. Reads were discarded if either the sequence preceding or following the homopolymer was less than 5bp in length (accounting for the GIAB slop used). All reads were then stacked into a matrix, according to their positional offset relative to the homopolymer, and error rate per pos-offset was computed.
The average error rate was computed for avidity sequencing runs and for publicly available data from multiple SBS instruments, for comparison. The differences of mismatch percentages, across all BED intervals, between AVITI™ and NovaSeq were plotted in a histogram and examples showing various percentiles within the distribution were chosen for display via IGV.

The interval-error.tsv and offset-error.tsv files can be found in the following directory: https://github.com/Elembio/AvidityManuscript2023/tree/main/data/homopolymer-error/GRCh38_SimpleRepeat_homopolymer_gt11_slop5
To compute the mismatch percent difference between avidity sequencing and SBS across homopolymer lengths, the four GIAB supplied homopolymer bed files were combined, and duplicates were removed (4to6, 7to11, gt11, gt20), producing a new bed file representing all homopolymer of size 4 to inf. The box plot shows median, quartiles, and the whiskers are 1.5*IQR.

For manuscripts utilizing custom algorithms or software that are central to the research but not yet described in published literature, software must be made available to editors and reviewers. We strongly encourage code deposition in a community repository (e.g. GitHub). See the Nature Portfolio guidelines for submitting code & software for further information.

Data

Policy information about availability of data

All manuscripts must include a data availability statement. This statement should provide the following information, where applicable:

- Accession codes, unique identifiers, or web links for publicly available datasets
- A description of any restrictions on data availability
- For clinical datasets or third party data, please ensure that the statement adheres to our policy

The avidity sequencing data sets described in the manuscript are available for download via the AWS CLI using the following command:
aws s3 ls --no-sign-request s3://avidity-manuscript-data/

Samples and FASTQ have been accessioned in SRA under BioProject PRJNA869673.

Bioinformatic tools and scripts can be found on the following github repo: https://github.com/Elembio/AvidityManuscript2023

Human research participants

Policy information about studies involving human research participants and Sex and Gender in Research.

Reporting on sex and gender	There were no human research participants in this study.
Population characteristics	There were no human research participants in this study.
Recruitment	There were no human research participants in this study.
Ethics oversight	There were no human research participants in this study.

Note that full information on the approval of the study protocol must also be provided in the manuscript.

Field-specific reporting

Please select the one below that is the best fit for your research. If you are not sure, read the appropriate sections before making your selection.

☒ Life sciences ☐ Behavioural & social sciences ☐ Ecological, evolutionary & environmental sciences

For a reference copy of the document with all sections, see nature.com/documents/nr-reporting-summary-flat.pdf

Life sciences study design

All studies must disclose on these points even when the disclosure is negative.

Sample size	Sequencing calibration studies were performed on 20 samples. Single cell studies were performed on multiple samples that generated consistent results, but a single example was used for this particular study. To determine k-mer errors, a million k-mers of each length were used to determine percent mismatch. For the homopolymer analysis, ~700,000 loci were used. For GiaB stratifications, we selected context classes with at least 100 variants.
Data exclusions	There was no data excluded (Filtered data is excluded from the sequencing runs).
Replication	We checked that all presented runs are representative by looking at no fewer than 20 sequencing runs. For analyses such as homopolymer and k-mer accuracy, sample size calculations are based on the number of relevant loci within a run. There were no failures to replicate.
Randomization	The study performed was validating first principles studies such as enzyme kinetics to validate the hypotheses of avidity chemistry, thus sample randomization would not be necessary. Sequencing data was performed on known samples and comparative metrics to known reference samples also obviates the need for randomization of the studies as the known reference samples are a widely known control.
Blinding	The study performed was validating first principles studies such as enzyme kinetics to validate the hypotheses of avidity chemistry, thus blind would not be necessary. Sequencing data was performed on known samples and comparative metrics to known reference samples also obviates the need for blind studies as the known reference samples are a widely known control.

Reporting for specific materials, systems and methods

We require information from authors about some types of materials, experimental systems and methods used in many studies. Here, indicate whether each material, system or method listed is relevant to your study. If you are not sure if a list item applies to your research, read the appropriate section before selecting a response.

Materials & experimental systems

n/a	Involved in the study	
☒	☐	Antibodies
☒	☐	Eukaryotic cell lines
☒	☐	Palaeontology and archaeology
☒	☐	Animals and other organisms
☒	☐	Clinical data
☒	☐	Dual use research of concern

Methods

n/a	Involved in the study	
☒	☐	ChIP-seq
☒	☐	Flow cytometry
☒	☐	MRI-based neuroimaging
